# Supplementary material for: Electrochemical Performance of Nitrogen Self-Doping Carbon Materials Prepared by Pyrolysis and Activation of Defatted Microalgae
Source: Molecules. 2023 Oct 26;28(21):7280. doi: 10.3390/molecules28217280 (PMC10648935; doi:10.3390/molecules28217280)
Supplement: Supplementary file 1 [file molecules-28-07280-s001.zip › molecules-2660429-supplementary.pdf]

Electrochemical performance of nitrogen self-doping carbon materials prepared by pyrolysis-activation of microalgae

Xin Wang [*, a], Lu Zuo [a], Yi Wang [a], Mengmeng Zhen [a], Lianfei Xu [a], Wenwen Kong [a], Boxiong Shen [b, c]

[a] Tianjin Key Laboratory of Clean Energy and Pollution Control, School of Energy and Environmental Engineering, Hebei University of Technology, P.R. China

[b] School of Chemical Engineering, Hebei University of Technology, P.R. China

[c] Hebei Engineering Research Center of Pollution Control in Power System, Tianjin, P.R. China, 300401

*Corresponding Author: Xin Wang

Address: Xiping Road No. 5340, Beichen District, Tianjin, P.R. China, 300401

Phone: 022-60435784

Fax: 022-60435784

E-mail: wangxinnh@hebut.edu.cn

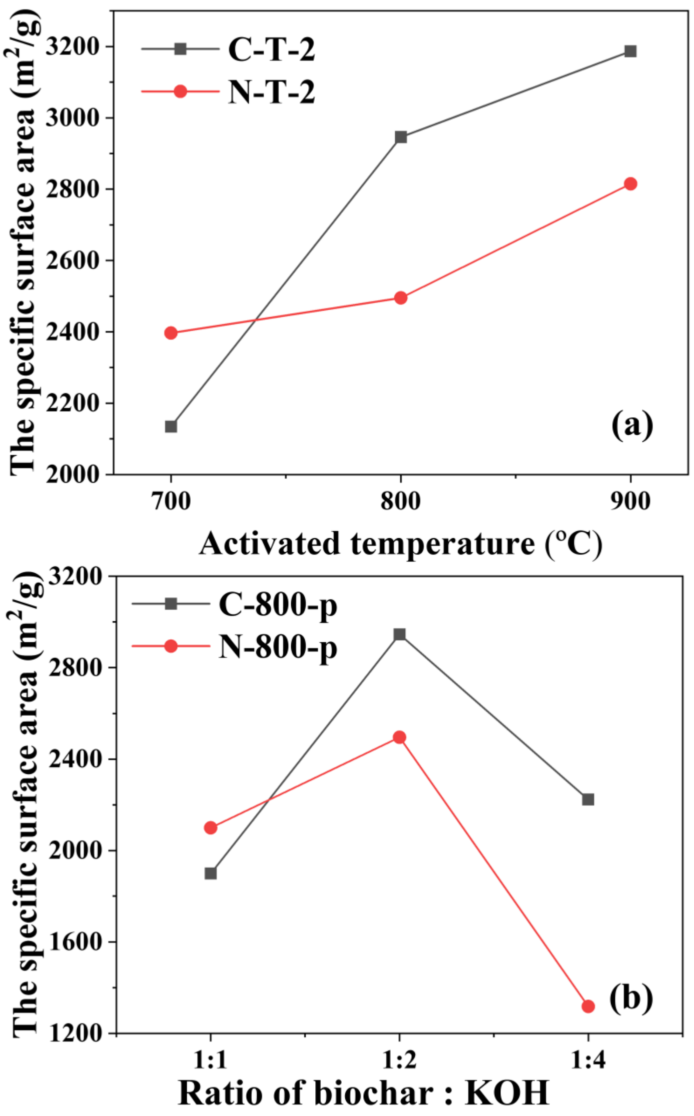

Figure S1 (a) When the ratio of biochar to KOH is 1:2, the relationship between temperature and specific surface area; (b) When the activated temperature is 800 °C, the relationship between the ratio of biochar to KOH and the specific surface area.

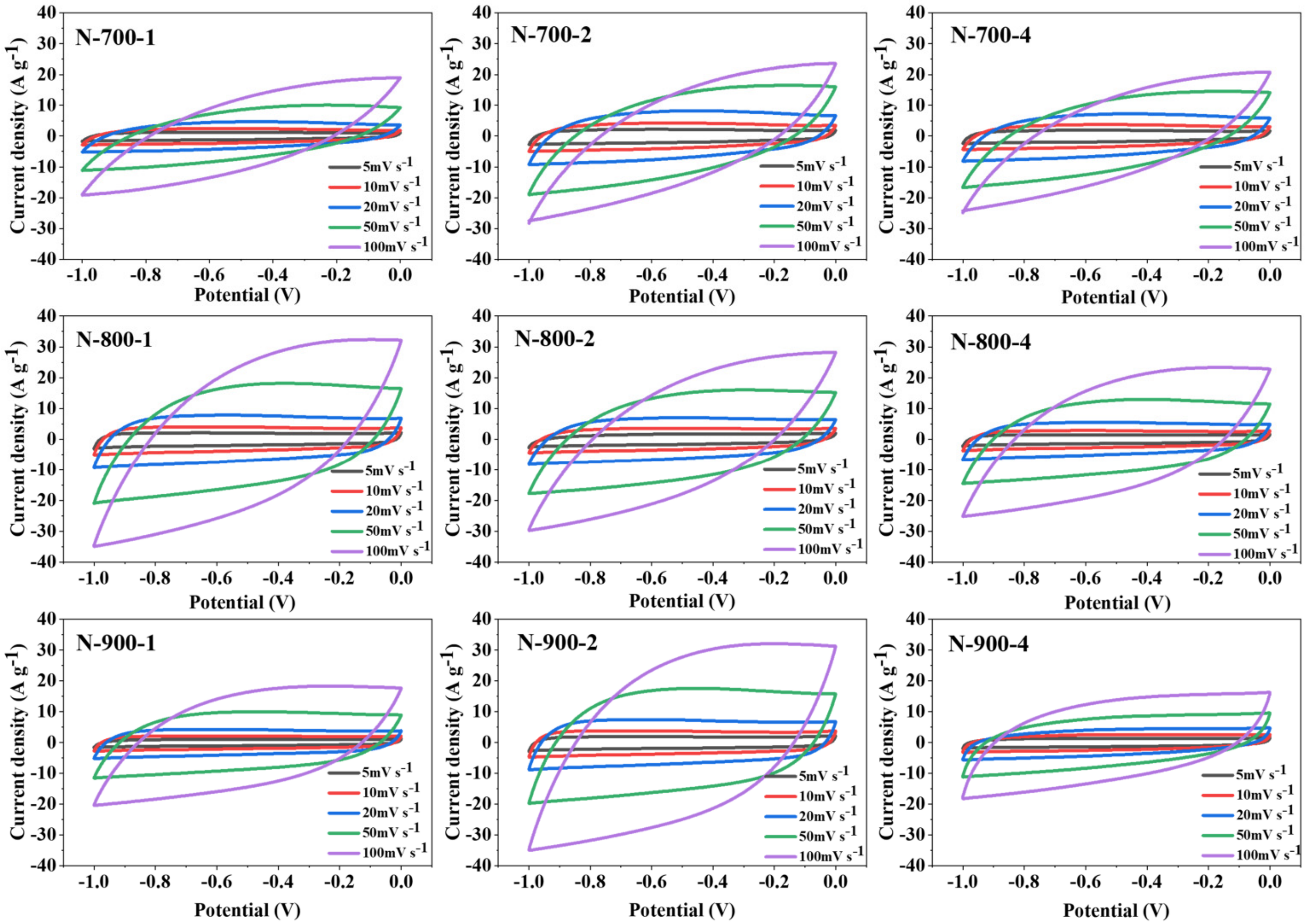

Figure S2 CV curves of all the *Nanochloropsis*-based N-doped activated carbon

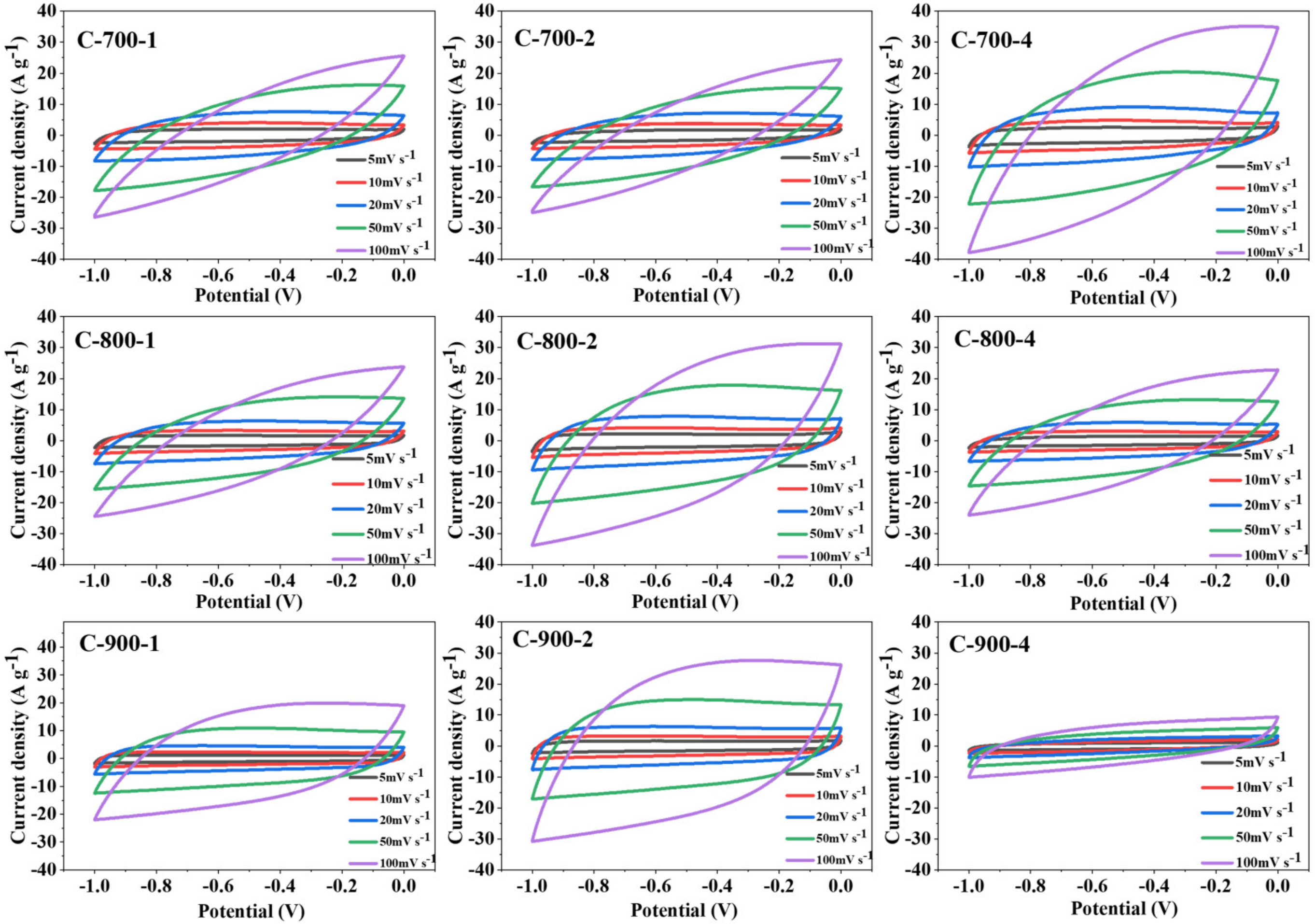

Figure S3 CV curves of all the *Chlorella*-based N-doped activated carbon

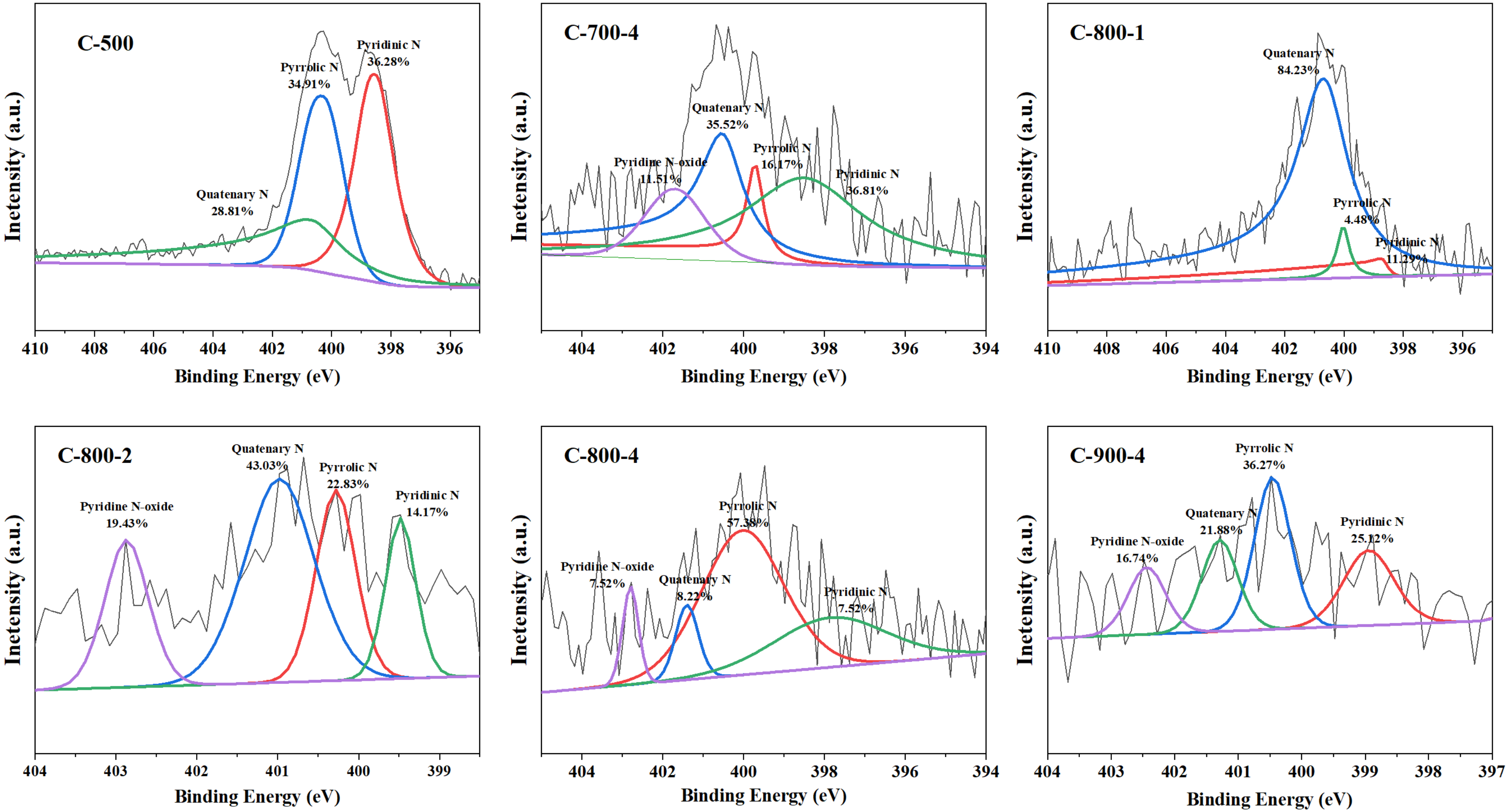

Figure S4 N 1 s spectra of *Chlorella*-based N-doped activated carbon

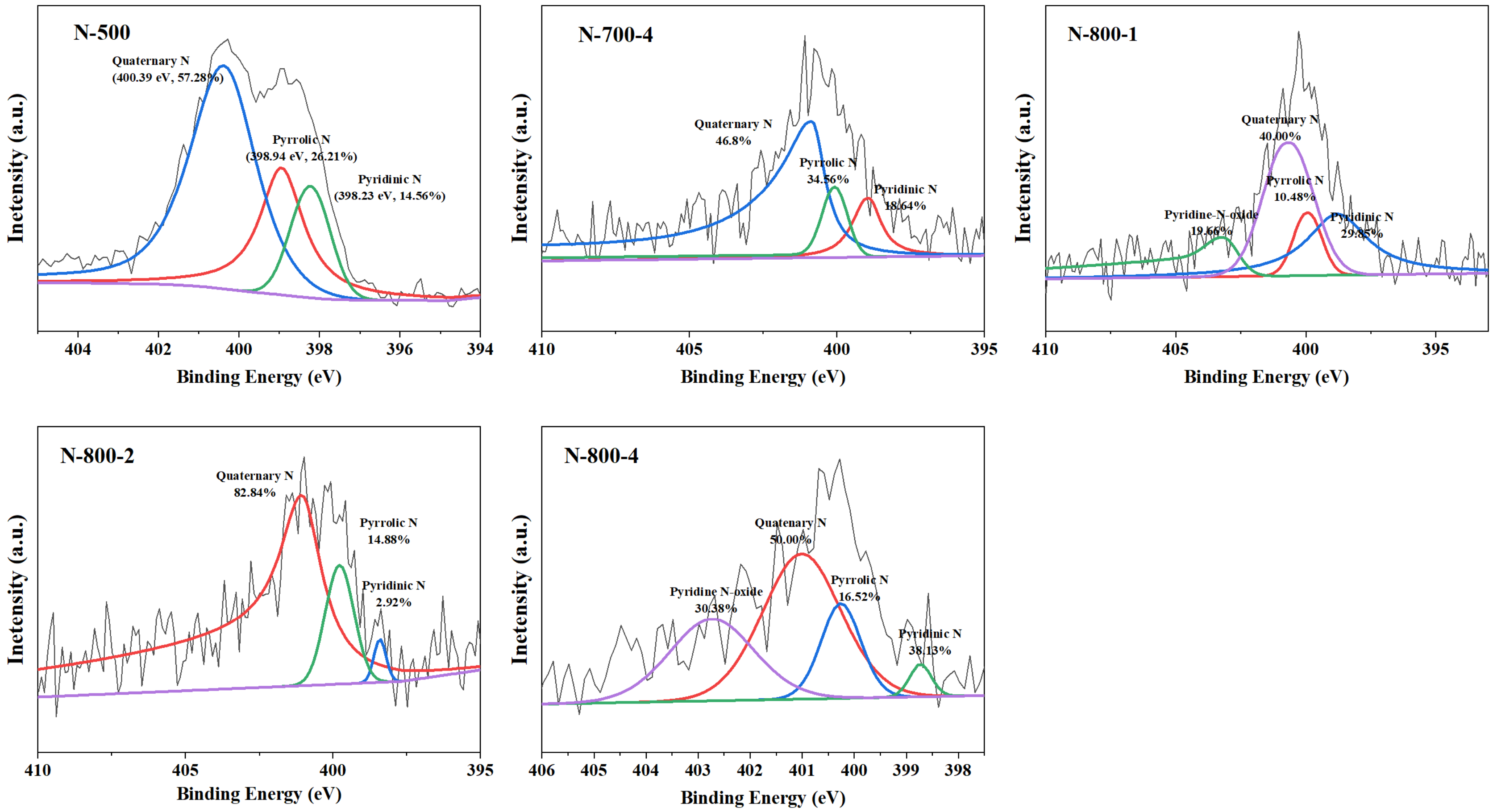

Figure S5 N 1 s spectra of *Nanochloropsis*-based N-doped activated carbon

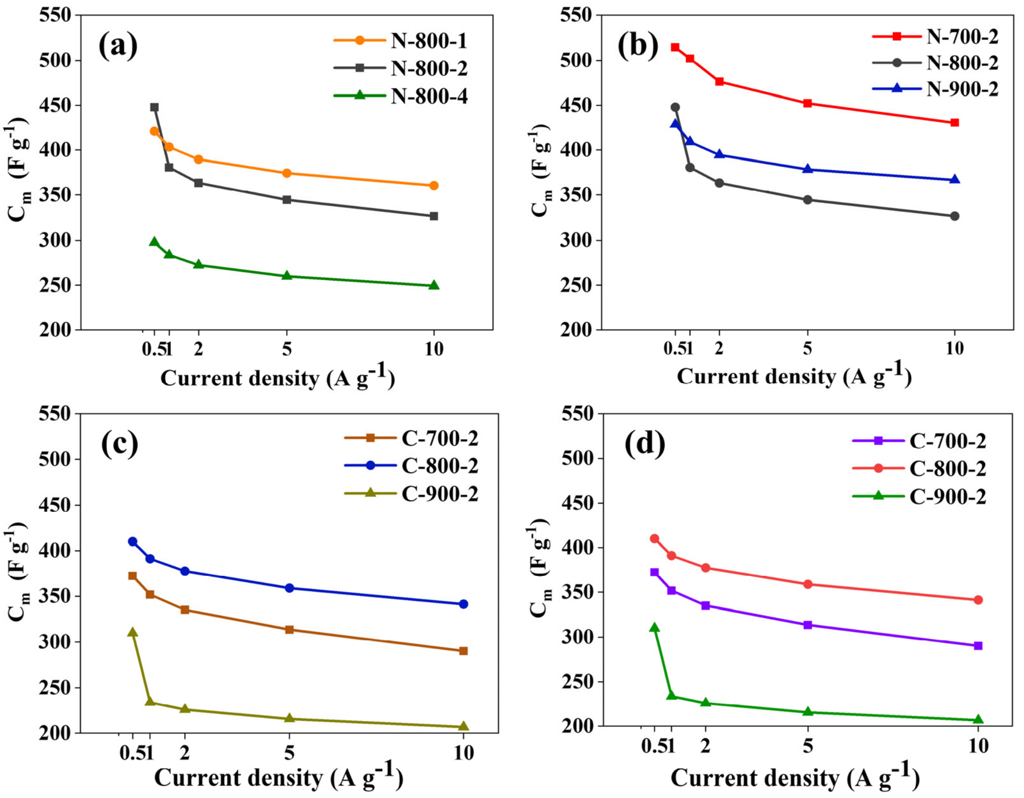

Figure S6 Specific capacitance of microalgae-based N-doped activated carbon from 0.5 to 10 A g^{-1}: (a) N-800-1, N-800-2, N-800-4; (b) N-700-1, N-700-2, N-700-4; (c) C-800-1, C-800-2, C-800-4; (d) C-700-2, C-800-2, C-900-2.

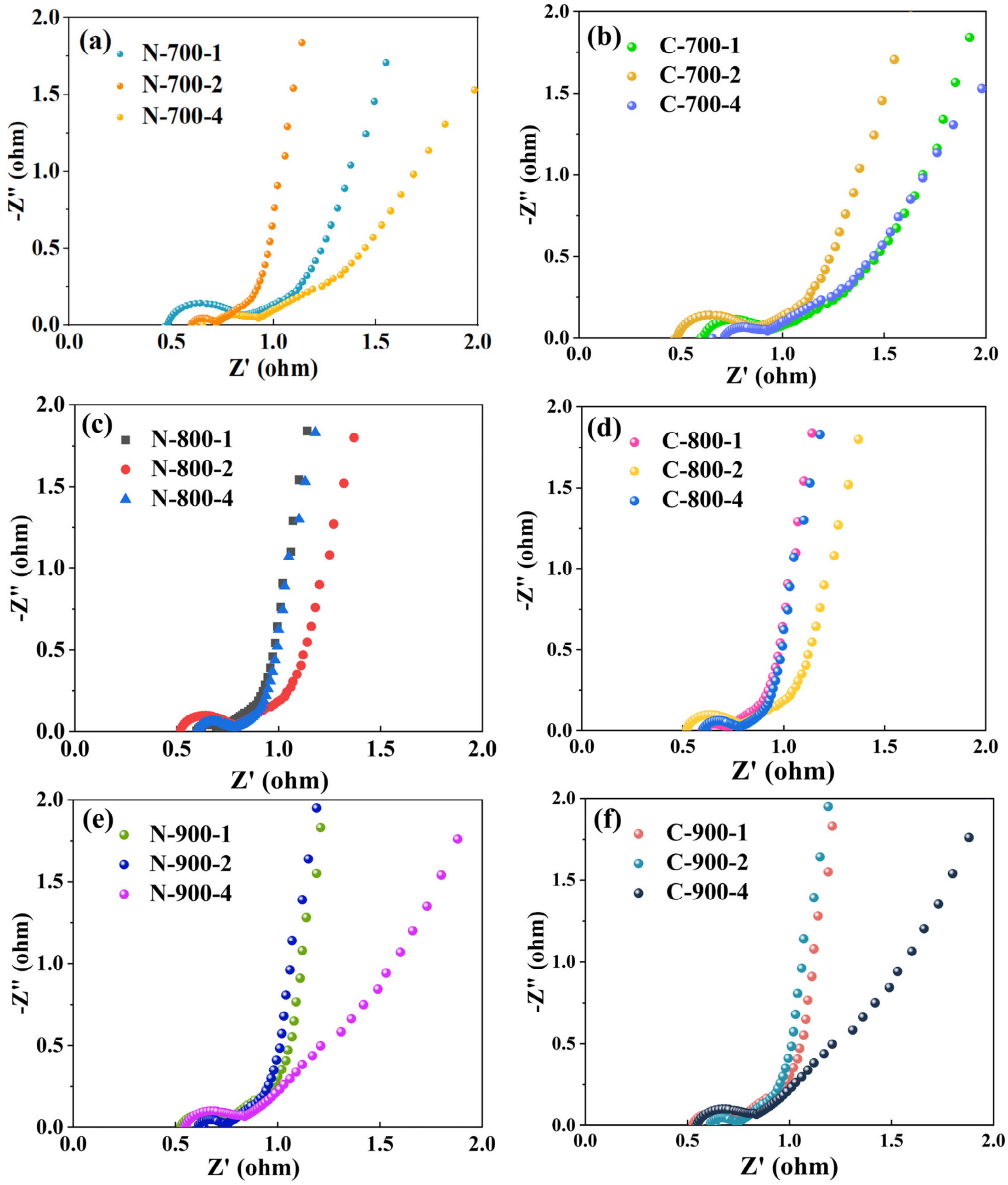

Figure S7 Nyquist plots of the microalgae-based N-doped activated carbon electrodes

Table S1 Composition of microalgae *Nanochloropsis* and *Chlorella*

Item	*Nanochloropsis*	*Chlorella*
Water (%)	2.2	4.0
Ash (%)	14.5	4.4
Lipid (%)	18.5	13.7
Protein (%)	39.5	58.9
Carbohydrate (%)	25.6	19.0
C (%)	42.35	49.56
N (%)	6.51	9.49
O (%)	27.19	24.77
H (%)	6.33	7.34
S (%)	0.92	0.44

Table S2 Profiling of amino acids in microalgae (mg/100 mg)

Item	*Nanochloropsis*	*Chlorella*
Aspartic acid	2.968	4.798
Threonine	1.632	2.260
Serine	1.555	2.123
Glutamine	4.714	7.964
Glycine	1.871	2.980
Alanine	2.661	4.606
Valine	1.897	2.678
Asparagine	0.644	1.029
Isoleucine	1.135	1.387
Leucine	3.192	4.960
Tyrosine	0.724	1.484
Phenylalanine	0.842	1.934
Histidine	1.012	1.713
Lysine	3.479	4.064
Arginine	0.584	1.049
Proline	2.970	2.928
Aspartic acid	4.371	3.080
Total	36.251	51.037

Table S3 Capacitance retention of microalgae-based N-doped activated carbon materials from 0.5 to 10 A g^{-1}

Sample	Capacitance retention (%)	Sample	Capacitance retention (%)
N-800-1	85.69	C-800-1	81.21
N-800-2	72.88	C-800-2	83.25
N-800-4	83.70	C-800-4	78.93
N-700-2	83.69	C-700-2	77.96
N-900-2	85.57	C-900-2	66.66

Table S4 Simulation results of equivalent circuit elements

Sample	R_s (Ω)	R_{ct} (Ω)	W(Ω)	C_d (F g^{-1})	R_{sum} (Ω) [a]
N-700-1	0.535	0.195	1.030	0.944	1.760
N-700-2	0.492	0.309	1.167	0.953	1.969
N-700-4	0.739	0.094	1.594	1.165	2.427
N-800-1	0.614	0.216	0.910	1.088	1.607
N-800-2	0.530	0.083	1.282	0.972	2.028
N-800-4	0.612	0.144	0.470	0.953	1.226
N-900-1	0.545	0.146	0.950	1.062	1.641
N-900-2	0.627	0.090	0.698	1.084	1.415
N-900-4	0.573	0.121	0.114	1.190	0.808
C-700-1	0.623	0.273	2.449	0.902	3.345
C-700-2	0.568	0.313	2.408	0.917	3.289
C-700-4	0.565	0.273	1.156	0.869	1.994
C-800-1	0.639	0.175	1.228	0.891	2.042
C-800-2	0.512	0.142	0.996	0.985	1.650
C-800-4	0.573	0.275	2.019	0.954	2.867
C-900-1	0.619	0.140	0.806	1.007	1.565
C-900-2	0.629	0.179	0.990	1.051	1.798
C-900-4	0.567	0.414	1.550	0.886	2.534

a: $R_{sum} = R_s + R_{ct} + W_t$
